# Hormonal Alterations in Individuals with Obesity After Metabolic Bariatric Surgery: A Narrative Review

**DOI:** 10.3390/medicina61101724

**Published:** 2025-09-23

**Authors:** Ioanna A. Anastasiou, Dimitris Kounatidis, Eleni Rebelos, Natalia G. Vallianou, Anastasios Tentolouris, Nikolaos Tentolouris, Maria Dalamaga, Irene Karampela

**Affiliations:** 1 Diabetes Center, First Department of Propaedeutic Internal Medicine, Medical School, National and Kapodistrian University of Athens, Laiko General Hospital, 11527 Athens, Greece; anastasiouiwanna@gmail.com (I.A.A.); dimitriskounatidis82@outlook.com (D.K.); elenirebelos@gmail.com (E.R.);; 2 Department of Pharmacology, National and Kapodistrian University of Athens, 11527 Athens, Greece; 3 Turku PET Centre, University of Turku, 20521 Turku, Finland; 4 Department of Clinical and Experimental Medicine, University of Pisa, 50134 Pisa, Italy; 5 First Department of Internal Medicine, Sismanogleio General Hospital, 15126 Athens, Greece; natalia.vallianou@hotmail.com; 6 Department of Biological Chemistry, National and Kapodistrian University of Athens, 15771 Athens, Greece; madalamaga@med.uoa.gr; 7 Second Department of Critical Care, Attikon General University Hospital, National and Kapodistrian University of Athens, 12462 Athens, Greece

**Keywords:** adjustable gastric band, metabolic bariatric surgery, body mass index, gut hormones, metabolic surgery, obesity, Roux-en-Y gastric bypass, sleeve gastrectomy

## Abstract

The gastrointestinal (GI) tract is increasingly recognized as an important regulator of energy balance and metabolism, extending beyond its traditional digestive functions. This review synthesizes current research on how modifications to the GI tract, particularly those induced by metabolic bariatric surgery (MBS), influence hormonal and physiological processes involved in glucose regulation and appetite control. MBS procedures, such as Roux-en-Y gastric bypass (RYGB) and sleeve gastrectomy (SG), induce significant and sustained weight loss, but also elicit adaptive morphological and functional changes within the intestines. These alterations include intestinal hypertrophy, increased mucosal surface area, changes in nutrient transit time, and modifications in enzyme activity. Such changes enhance the secretion of key gut hormones, including glucagon-like peptide 1 (GLP-1) and peptide YY (PYY), which play vital roles in promoting insulin secretion, suppressing appetite, and improving blood glucose regulation. The benefits stem from the exposure of undigested nutrients to different intestinal segments, which stimulates enteroendocrine activity and positively influences systemic metabolism. These hormonal adaptations contribute significantly to the metabolic improvements observed post-surgery, independent of weight loss alone. Understanding how gut structural and functional changes drive hormonal responses provides valuable insights into the mechanisms underlying the success of MBS. Moreover, elucidating these processes may facilitate the development of less invasive therapies that mimic the metabolic benefits of surgery. Ultimately, this research advances our understanding of gut-mediated regulation of energy and glucose homeostasis and holds promise for improving treatment strategies for obesity and related metabolic disorders.

## 1. Introduction

According to the World Health Organization (WHO), obesity is defined as a body mass index (BMI) greater than 30 kg/m^2^ [1]. In developed countries, obesity is the second leading preventable cause of death after smoking, and it is now recognized as a global health emergency [2]. Obesity is frequently associated with multiple comorbidities, the most prominent of which include type 2 diabetes (T2D), hypertension, cardiovascular disease (CVD), and chronic kidney disease (CKD). Additionally, it often coexists with obstructive sleep apnea (OSA), osteoarthritis, and depression, and it is significantly linked to increased cancer risk [3,4,5]. Given the substantial impact of obesity on human health, the development and implementation of effective treatment strategies has become urgent. Pharmacotherapy plays a central role in obesity management, particularly with the emergence of incretin-based therapies such as glucagon-like peptide 1 (GLP-1) receptor agonists and dual glucose-dependent insulinotropic peptide (GIP)/GLP-1 receptor agonists. Among these, tirzepatide has emerged as a prominent therapeutic agent. However, when pharmacological approaches fail to achieve adequate metabolic control or weight loss, metabolic bariatric surgery (MBS) is indicated [6].

MBS induces weight loss primarily through alterations in nutrient absorption and satiety signaling [7]. According to the 1991 National Institutes of Health (NIH) guidelines, individuals with a BMI ≥40 kg/m^2^, or ≥35 kg/m^2^, with significant obesity-related comorbidities, should be considered for MBS [8]. While these guidelines are still widely applied, accumulating evidence supports the consideration of bariatric procedures in individuals with T2D and a BMI between 30 and 35 kg/m^2^ when glycemic control is insufficient despite optimized medical therapy. Common bariatric procedures include adjustable gastric banding (AGB), Roux-en-Y gastric bypass (RYGB), and sleeve gastrectomy (SG) [9]. Biliopancreatic diversion with duodenal switch (BPD-DS), once more commonly performed, is now less frequently utilized due to its potent malabsorptive effects, which can significantly compromise individuals’ quality of life [10]. One-anastomosis gastric bypass (OAGB) features a longer gastric pouch and a single gastrojejunostomy [11]. Irrespective of the surgical technique, robust data support the superior efficacy of MBS over non-surgical interventions in promoting sustained weight loss and improving glycemic outcomes in T2D [8].

The hormonal changes induced by MBS are complex and central to its metabolic benefits. In the stomach, hormones like gastrin decrease post-surgery, affecting gastric acid and mucosal health, while ghrelin, mainly produced in the fundus, drops significantly, reducing appetite and promoting weight loss [12]. Obestatin, derived from the same precursor, is also lower in obesity and after gastrectomy. In the intestines, incretins such as GLP-1 and peptide YY (PYY) increase markedly, enhancing insulin secretion, satiety, and glucose regulation, key factors in T2D remission. GIP responses vary, but generally decline after bypass procedures, influencing insulin release. Cholecystokinin (CCK) levels tend to elevate postprandially, further suppressing appetite. Hormones like secretin and neurotensin are altered depending on the procedure, affecting digestive processes. Pancreatic hormones, including insulin and glucagon, show improved β-cell function and insulin sensitivity, reflected in decreased homeostasis model assessment insulin resistance (HOMA-IR). Additionally, fibroblast growth factors (FGF-19 and FGF-21) are modulated, impacting glucose, lipids, and mitochondrial function, supporting metabolic remission. These hormonal shifts work synergistically to promote weight loss, enhance insulin sensitivity, and resolve obesity-related complications [12].

This narrative review aims to synthesize current evidence on the metabolic effects of various MBS procedures, focusing on elucidating their hormonal, physiological, and clinical mechanisms and evaluating their effectiveness in treating obesity and T2D.

## 2. Literature Search

For this review, a comprehensive search of the PubMed database was conducted using the terms “metabolic surgery” and “hormones.” To focus on current evidence, the search was limited to publications from 2000 to August 2025, yielding 1989 articles. The selection process prioritized research articles, randomized controlled trials, and meta-analyses to ensure a rigorous and evidence-based synthesis. Furthermore, the reference lists of the included articles were manually searched to identify additional relevant studies. Due to the large volume of literature, the authors acknowledge that this review may not encompass all potentially relevant publications.

## 3. Metabolic Bariatric Surgery: From Weight Loss to Metabolic Transformation

Although the earliest methods of MBS were described in the early 1950s, the metabolic benefits of MBS became increasingly recognized in the mid-1990s, revealing that its positive effects extend far beyond mere weight reduction. Improvement in metabolic health occurs through physiological changes, including alterations in hormone regulation and gut microbiota, which collectively enhance insulin sensitivity and overall metabolic function [13]. Subsequently, and thanks to the significant weight loss, several obesity-related comorbidities such as T2D, hypertension, and OSA are improved.

A multicenter review examined the use of metabolic and MBS as a preparatory step for patients with obesity and primary neoplasia deemed high-risk for direct surgical treatment [14,15]. Among 37 patients (median age 52, BMI 49.1 kg/m^2^), most underwent sleeve gastrectomy, with no MBS-related complications reported over an average follow-up of 4.3 years. The majority (83.8%) subsequently underwent neoplastic surgery, achieving significant weight loss (BMI decline from 49.9 to 39.7 kg/m^2^) within approximately six months. There were two procedure-related mortalities. The findings suggest that MBS can be a feasible strategy for reducing surgical risks and improving outcomes in patients with low-grade, less aggressive tumors, emphasizing the importance of personalized, multidisciplinary care [14,15].

## 4. Principal Techniques of Metabolic Bariatric Surgery

**Figure 1:**
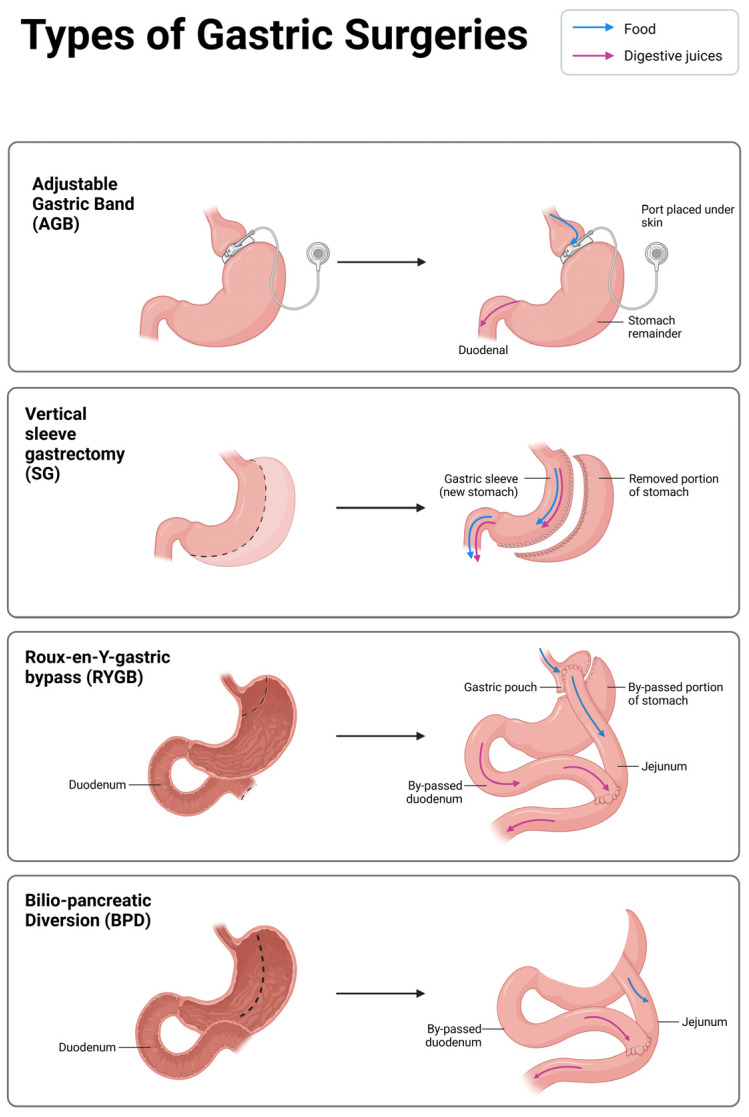
Principal techniques of metabolic bariatric surgery highlighting their structures and the flow of food and digestive juices. Created in BioRender. Anastasiou, I.A. (2025) https://BioRender.com/deiuy3g (accessed on 7 September 2025).

Various surgical techniques have been employed in MBS, including adjustable gastric banding (AGB), Roux-en-Y gastric bypass (RYGB), biliopancreatic diversion (BPD), and sleeve gastrectomy (SG) (Figure 1).

### 4.1. Adjustable Gastric Band (AGB)

The only procedure that uses an implanted foreign body is AGB, which was first used in 1986 [16]. AGB is a surgical procedure designed to facilitate weight loss by dividing the stomach into two sections and creating a small-volume pouch above the gastric band, without requiring gastric transection or stapling. The system consists of an inflatable collar and a silicone belt. The degree of constriction provided by the band can be modified by injecting saline into the collar via a subcutaneous implantable chamber connected through tubing [17]. However, recent data from the International Federation for the Surgery of Obesity and Metabolic Disorders (IFSO) indicates a notable decline in the utilization of AGB for obesity management. Specifically, the prevalence of AGB procedures has plummeted from 42.3% to 11.4% over the past decade, beginning in 2008. This shift occurs despite the initial enthusiasm surrounding the procedure, suggesting that healthcare professionals and individuals may be favoring alternative bariatric options that offer more effective long-term outcomes [16].

### 4.2. Roux-en-Y Gastric Bypass (RYGB) and Bilio-Pancreatic Diversion (BPD)

The RYGB was first developed as an open procedure in 1966 by Dr. Edward Mason and Dr. Chikashi Ito. The laparoscopic approach was later introduced in 1994, marking a significant advancement in minimally invasive MBS [18]. The essential steps of the gastric bypass procedure include the creation of an approximate 20 to 30 mL gastric pouch, creation of a gastrojejunal anastomosis, and creation of a jejunojejunostomy between the Roux and the biliopancreatic limb. The desired length of the Roux limb and the biliopancreatic limb varies, but most agree on lengths of 75 to 150 cm and 40 to 50 cm, respectively [18]. RYGB is still the most traditional and widely used surgical procedure for obesity.

Scopinaro first described BPD in 1979 [19]; it involved a distal gastrectomy, to which the final 250 cm of the small intestine was anastomosed using a 50 cm common loop. Hess and Marceau modified BPD in North America at the end of the 1980s by dividing the duodenum about three centimeters from the pylorus (a procedure known as BPD with duodenal switch, or BPD-DS) and performing a gastrectomy in the shape of a vertical tube (similar to SG) in order to anastomose the 250 cm of the distal small intestine, this time using a standard 100 cm loop. The frequency of BPD-DS has never surpassed 1–2% of MBS procedures, despite the fact that this most recent version has forced the BPD, as explained by Scopinaro [19].

### 4.3. Sleeve Gastrectomy (SG)

SG was first applied to individuals with the most severe obesity in the early 2000s. With a growing growth curve, however, SG alone has swiftly gained so much popularity that it now accounts for over half of all MBS performed globally. It involves dividing the stomach wall around a calibration tube with a variable diameter (32 to 50 Fr) and stapling it to form a gastric tube along the lesser gastric curvature. The majority of the stomach is removed without breaking the flow. Despite being a relatively easier-to-perform intervention compared to RYGB, not all individuals with severe obesity are good candidates for SG. For example, the presence of gastroesophageal reflux, a common condition in the general population and in persons with obesity, is a relative contraindication for SG, as reflux can worsen following this intervention [20].

### 4.4. One-Anastomosis Gastric Bypass (OAGB)

In recent years, a variation of classical RYGB has been developed. Here, the gastric pouch is a bit longer compared to the traditional gastric pouch of RYGB, and an end-to-side gastrojejunostomy is created, thus allowing for a single anastomosis (rather than two, as in classical RYGB) [21].

Because the mechanisms of surgery differ from those of lifestyle and/or medical therapy, there is a great deal of potential for using combinatorial strategies to improve patient outcomes over the long term [22].

## 5. Gastrointestinal Hormones and Metabolic Bariatric Surgery

Gastrointestinal (GI) hormones are a diverse group of peptides synthesized and secreted by specialized cells within the gastrointestinal tract, which is considered the body’s largest endocrine organ due to its extensive capacity to produce and release these regulatory peptides [23]. These hormones are predominantly released by endocrine cells located within the mucosal lining of various segments of the GI tract, including the stomach, small intestine (duodenum, jejunum, ileum), and colon. In addition, paracrine cells such as enterochromaffin cells and neurons situated within the enteric nervous system contribute to the localized and systemic release of these peptides. The secretion of GI hormones is highly organized, with specific cell types and hormone profiles distributed systematically along the digestive tract in a manner that aligns with the physiological functions of each segment. For example, G cells in the antrum and duodenum secrete gastrin, which regulates gastric acid secretion, while I cells in the duodenum produce CCK, which stimulates pancreatic enzyme release and gallbladder contraction. L cells in the ileum and colon release hormones like GLP-1 and PYY, which are important for appetite regulation and glucose homeostasis. The release of these hormones is tightly regulated by various stimuli such as the presence of nutrients (fats, amino acids, glucose) in the lumen, neural inputs from the vagus nerve, and local paracrine signals, creating a highly coordinated system that influences digestion, nutrient absorption, and metabolic regulation throughout the entire gastrointestinal tract [23].

### 5.1. Stomach Hormones

#### 5.1.1. Gastrin

Gastrin is a peptide hormone (34 amino acids) that stimulates parietal cells in the stomach to secrete gastric acid and is involved in gastric motility [24,25]. Secreted by G cells located in the duodenum, pancreas, and pyloric antrum of the stomach, gastrin promotes the release of histamines from enterochromaffin-like cells by binding to CCK B receptors. It also facilitates the insertion of K+/H+ ATPase pumps into the apical membrane of parietal cells, thereby enhancing hydrogen ion secretion into the stomach lumen. The release of gastrin is triggered by the presence of peptides in the stomach, and it also plays a role in appetite suppression. Importantly, gastrin is not a single molecular entity but rather a family of peptides with varying lengths and biological activities [24,25].

Several studies have investigated the complex physiological and hormonal changes following MBS such as RYGB and SG, demonstrating that postprandial gastrin levels typically decrease after RYGB, both in the early two weeks and throughout the first-year post-operation [26,27]. A study [28] demonstrated that individuals with stomal ulcers post-RYGB experienced significantly elevated gastric acid exposure, with a median of 69% of the time with pH below 4, compared to 20% in controls. These findings implicate acid exposure as a potential factor in ulcer formation, independent of fistula presence [28]. Another prospective study [29] assessed the effects of laparoscopic AGB, showing an average excess weight loss of 45.7% over 12 months, accompanied by a sharp decrease in leptin levels from 19.7 ng/mL to 6.9 ng/mL (*p* < 0.001). Despite weight loss, hormones like ghrelin, PP, insulin, GIP, GLP-1, gastrin, and pepsinogen I remained stable, with only transient changes in pepsinogen II, indicating preservation of overall gastrointestinal endocrine function [29].

Research [30] comparing gastrin secretion and metabolic responses found that RYGB was associated with significantly lower serum gastrin levels and reduced glucose area under the curve (AUC) during meal testing (*p* = 0.013), implying altered hormonal regulation and enhanced glycemic control. In contrast, SG showed a trend toward increased gastrin secretion without reaching statistical significance (*p* = 0.091), and neither procedure caused hypergastrinemia. These variations highlight that the type of MBS differentially impacts gastric hormone secretion and mucosal function [30]. Finally, histological analysis [31] of the excluded stomach after RYGB revealed increased cell proliferation (higher ki-67 index) and decreased apoptosis (lower caspase-3 expression), along with a significant reduction in gastrin-positive cells (29.6 ± 7.9 vs. 55.5 ± 11.7; *p* = 0.0003). These findings suggest active mucosal remodeling, through increased proliferation and decreased programmed cell death, which may influence long-term gastric mucosal integrity [31]. Biomarker studies [32] further indicated that 80% of SG individuals had low protein glycation index (PGI) levels (<30 µg/L), a marker of gastric atrophy, mainly affecting the fundic mucosa. These individuals also exhibited elevated fasting G17 levels (13.9 ± 17.2 pmol/L), with 40% exceeding the normal upper limit, reflecting the impaired acid-secreting capacity of the stomach’s fundic region [32].

These studies collectively demonstrate that MBS induces significant structural, hormonal, and functional changes within the gastrointestinal tract. RYGB tends to elevate gastric acid exposure, promote mucosal remodeling, and decrease gastrin secretion, while SG more profoundly impairs fundic gland function and acid secretion, as reflected by low PGI and elevated G17 levels. Understanding these alterations is important for optimizing postoperative management, predicting long-term risks, and tailoring treatment strategies to ensure patient safety and effective metabolic outcomes.

#### 5.1.2. Ghrelin

A 28-amino acid peptide that is mostly secreted in the stomach, ghrelin promotes the release of growth hormone (GH) and appetite [33,34]. The word “ghre,” which means “grow” in Proto-Indo-European languages, is the root of the name “ghrelin,” referring to its capacity to promote the release of growth hormone. Although ghrelin was first identified as a stomach-derived hormone that acts through hypothalamic circuits to regulate energy balance, hunger, and meal initiation, it is now evident that it also plays a part in motivated reward-driven behaviors by activating the so-called “cholinergic–dopaminergic reward link”. This reward link consists of a cholinergic input that originates mainly from the laterodorsal tegmental area and a dopamine projection from the ventral tegmental area (VTA) to the nucleus accumbens. The cholinergic–dopaminergic reward link is activated when ghrelin is administered into the VTA, which implies that ghrelin may raise the incentive value of motivated behaviors like reward-seeking behavior (also known as “wanting” or “incentive motivation”) [33,34].

Furthermore, mice and rats that receive ghrelin injections directly into their brain ventricles or the VTA consume more alcohol and rewarding foods [35]. Ghrelin receptor antagonists have been shown in rodent studies to have positive effects on reducing the intake of palatable foods, decreasing calorie-dense food preferences, and suppressing food reward and food-motivated behavior. Additionally, they have been demonstrated to lower alcohol intake and inhibit amphetamine-, cocaine-, and alcohol-induced rewards. Additionally, changes in the ghrelin receptor and pro-ghrelin genes have been linked to obesity, bulimia nervosa, smoking, heavy alcohol use, and increased weight gain in alcohol-dependent people. Therefore, the central ghrelin signaling system connects neurobiological circuits involved in food and chemical drug reward; substances that either directly or indirectly inhibit this system become promising candidate medications for treating substance use disorders and preventing problematic overeating that results in obesity [33,34].

In humans, several studies have investigated how MBS affects ghrelin levels and weight regulation. One study [36] observed that in individuals with obesity following a dietary regimen, plasma ghrelin levels increased significantly during the 24-h cycle, especially before meals, and the AUC for ghrelin increased by 24% as body weight decreased by 17% (*p* = 0.006). Conversely, in RYGB individuals, ghrelin levels were 77% lower than in controls and 72% lower than in matched controls with obesity, despite a 36% weight loss, with no normal food-related diurnal fluctuations, indicating a lasting suppression of ghrelin secretion linked to the surgical bypass of ghrelin-producing cells in the fundus [36]. Another study [37] compared weight loss induced by AGB, RYGB, and dietary treatment in 24 matched men, finding that only RYGB caused a significant decrease in fasting plasma ghrelin concentrations (from 424.6 ± 32.8 pg/mL to 131.4 ± 13.5 pg/mL; *p* < 0.001), despite similar weight loss across groups. Notably, even in individuals who underwent total gastrectomy, which completely removes ghrelin-producing tissue, ghrelin levels remained low, indicating that RYGB’s reduction in ghrelin is due to bypassing the fundus rather than a secondary effect of weight loss or improved insulin sensitivity [37].

A further comparison study [36] involving individuals who achieved comparable weight loss through AGB, RYGB, or biliopancreatic diversion showed that RYGB individuals had significantly lower fasting plasma ghrelin levels (average 117 ± 34 pg/mL) compared to both banding (480 ± 78 pg/mL) and BPD (406 ± 86 pg/mL), with no significant differences in BMI, fat mass, or metabolic markers. This suggests that the degree of fundus bypass or removal directly influences ghrelin secretion, with RYGB producing a more substantial suppression due to the anatomical disruption of the ghrelin-producing cells [38]. A systematic review and meta-analysis of 7 studies assessing the impact of SG on ghrelin levels also showed that SG leads to a reduction in ghrelin levels [39]. A study examined long-term hormonal responses in patients more than seven years after SG and BPD/RYGB dividing them into groups based on weight maintenance or regain [40]. The results showed that fasting levels of ghrelin, GLP-1, and PYY did not differ significantly between groups, and postprandial hormone responses largely remained similar. Notably, SG patients in the weight-maintaining group experienced greater suppression of ghrelin after a meal, while post-meal PYY levels were lower in the weight-maintenance group regardless of surgery type. Interestingly, higher PYY levels correlated with increased BMI, suggesting that these hormonal patterns are more reflective of ongoing weight changes, rather than being primary causes of weight regain. Overall, the data do not support the hypothesis that long-term weight regain is due to unfavorable gastrointestinal hormone secretion, highlighting the complexity of the factors involved in weight maintenance after bariatric surgery [40].

A literature review using PubMed and Google Scholar examined the impact of metabolic surgeries, especially gastric fundus resection via SG and modified laparoscopic gastric bypass with fundus resection (RYGB), on ghrelin levels and glucose regulation [41]. Of the numerous studies, 14 human investigations met the inclusion criteria. The findings indicate that reduced ghrelin levels after fundus resection may help early glycemic improvements, often before significant weight loss. However, long-term evidence on ghrelin’s role in glucose homeostasis is limited and inconclusive, highlighting the need for further research [41].

Collectively, these studies highlight that MBS involving bypass or removal of the gastric fundus significantly reduces circulating ghrelin levels, independent of weight loss or metabolic improvements. This suppression of ghrelin likely contributes importantly to decreased appetite and sustained weight loss, reinforcing the idea that anatomical disruption of ghrelin-producing cells plays a crucial role in the long-term success of certain bariatric procedures.

#### 5.1.3. Obestatin

Several mammals, including humans, have specialized epithelial cells in their stomachs and small intestines that produce the hormone obestatin (23 amino acids) [42,43,44]. Though its impact on food intake is still debatable, obestatin was first discovered to be an anorectic peptide. The same gene that produces the peptide hormone ghrelin also encodes the hormone obestatin. Ghrelin breaks down into proghrelin, which breaks down into unacylated ghrelin (28 amino acids) and acylated ghrelin. Presumably, obestatin is cleaved from C-ghrelin [42,43,44]. Limited research has examined the role of obestatin in obesity and sustained weight management. One study [45] reported that subjects with severe obesity had significantly lower fasting obestatin levels compared to lean controls (17.2 ± 2 vs. 27.8 ± 4 pg/mL, *p* = 0.03). Individuals who underwent gastrectomy showed a decrease in obestatin levels, though it was not statistically significant, and the obestatin/ghrelin ratio was higher in gastrectomy individuals (0.05 ± 0.009) than in lean and controls with obesity, indicating altered hormone balance. The study also noted that meal intake did not significantly change obestatin levels, suggesting that obestatin secretion remains stable after eating and may originate from sources outside the stomach [45]. Another investigation [46] evaluated ghrelin and obestatin in individuals with obesity who achieved long-term weight loss through either RYGB or lifestyle intervention. The results showed that RYGB individuals had higher fasting levels of both ghrelin and obestatin compared to preoperative controls, whereas the lifestyle group did not. Post-meal suppression of ghrelin was observed in all groups, but obestatin levels did not change significantly during the postprandial period. However, the ghrelin/obestatin ratio remained similar across all groups, suggesting that these hormones are regulated independently post-surgery [46].

Overall, in terms of appetite regulation, obestatin behaves in the opposite way compared to ghrelin, and thus far the evidence indicates that plasma obestatin levels are lower in individuals with obesity compared to lean controls, supporting a potential role in long-term body weight regulation. Obestatin levels tend to decrease following gastrectomy, which may influence appetite and energy balance beyond gastric hormonal production.

### 5.2. Pancreatic Hormones

#### 5.2.1. Insulin

Pancreatic islet β-cells synthesize insulin, a 51-amino-acid peptide hormone that plays a central role in regulating protein, fat, and carbohydrate metabolism by promoting glucose uptake, particularly in skeletal muscle, liver, and adipose tissue [26,47].

Several studies have investigated how MBS improves glucose metabolism and hormonal responses related to T2D. One study [48] assessed the early and late effects of MBS on insulin sensitivity and β-cell function. 35 individuals with T2D (23 RYGB, 12 SG) were studied undergoing mixed-meal testing before, 15 days after, and one-year post-surgery. The results showed long-term improvements in insulin sensitivity, which is closely linked to weight loss (*p* < 0.001), while β-cell glucose sensitivity improved both early and late after surgery (*p* < 0.005). Meal responses showed decreased pancreatic polypeptide (PP) in the early post-RYGB period, increased glucagon and GLP-1 at both time points, and elevated PYY at one year. Preoperative β-cell sensitivity and meal-stimulated GLP-1 responses predicted T2D remission [48]. A second study [49] assessed 12 T2D and 15 non-diabetic subjects with obesity before and one year after surgery using mixed-meal testing with double-tracer techniques. Postoperatively, glucose and insulin levels declined sharply, and insulin sensitivity improved with approximately 30% weight loss. β-cell glucose sensitivity doubled but remained below normal, indicating partial recovery. The rapid decline in plasma glucose and insulin, along with relative hyperglucagonemia, led to less suppressed endogenous glucose production during the meal, highlighting altered glucose regulation primarily driven by bypass surgery affecting hormonal responses [49].

Another study followed 10 T2D and 10 glucose-tolerant individuals with obesity pre- and post-RYGB at 1 week, 3 months, and 1 year [50]. Early after surgery, basal hepatic glucose production decreased, hepatic insulin sensitivity improved, and insulin clearance increased significantly, especially in T2D individuals, pointing to early liver adaptations. Later, peripheral insulin uptake and secretion improved, particularly in response to oral glucose, emphasizing that gut anatomy changes predominantly modulate postprandial insulin responses and glucose disposal [50]. A further study tracked 20 individuals after SG at 7, 30, and 90 days [51]. Subjects exhibited rapid reductions in BMI, insulin, and HOMA-IR, indicating swift improvements in insulin sensitivity. Early declines in fasting glucose and insulin levels correlated with weight loss, highlighting the rapid metabolic benefits that precede full weight stabilization [51]. Lastly, a comprehensive study compared three surgical procedures and dietary intervention over three years. All surgical groups exhibited significant and sustained decreases in HOMA-IR, with increases in PYY and adiponectin during the weight stability phase [52]. These hormones contributed to ongoing insulin sensitivity improvements independent of weight, suggesting that hormonal shifts post-surgery play a key role in long-term metabolic health [52].

Overall, MBS induces rapid and durable improvements in β-cell function, insulin sensitivity, and hormonal regulation, driven by anatomical changes affecting gut hormone secretion and liver function. Early adaptations in the liver and hormonal responses are crucial for achieving and maintaining diabetes remission and metabolic health.

#### 5.2.2. Glucagon

Alpha cells of the pancreatic islets of Langerhans, which are found in the endocrine section of the pancreas, produce the 29-amino acid peptide hormone, known as glucagon [53,54]. Insulin from the nearby β-cells suppresses or regulates its production. By encouraging glycogenolysis and gluconeogenesis, glucagon, which is also released during fasting, raises blood sugar levels [53,54]. The impact of MBS on circulating glucagon levels has been evaluated in relatively few studies.

A study examined the early effects of RYGB on hormonal and metabolic parameters in individuals with T2D [55]. Prior to and after RYGB, 10 individuals (BMI: 39.7 ± 1.9 kg/m^2^) underwent assessments, including plasma measurements of insulin, glucose, glucagon, and GLP-1, alongside a meal test. Notably, seven days post-surgery, the HOMA-IR index significantly decreased from 7.8 ± 5.5 to 2.6 ± 1.7, with a modest, non-significant weight reduction (*p* < 0.05). Hormonal responses showed that glucagon and glucose levels gradually declined during the meal, while insulin and GLP-1 peaked at 30 min. These changes persisted and intensified over time, with improvements in β-cell function and a positive correlation between insulin and GLP-1 levels (*p* = 0.000). Overall, RYGB appears to induce early hormonal adaptations that support improved glycemic control beyond weight loss [55]. In a separate study, Farey et al. investigated the effects of SG on fasting hormonal levels and non-esterified fatty acids (NEFA) in individuals with obesity, comparing the results to non-obese controls [56]. Using multiplex bead-based assays, nine hormones and NEFA were measured at baseline and three months post-operation in 11 individuals. Individuals with obesity exhibited lower ghrelin, GIP, and resistin, but higher C-peptide, insulin, and leptin compared to controls. Post-SG, BMI decreased from 42.5 ± 6.47 to 35.2 ± 5.14 kg/m^2^ (42% excess weight loss, *p* < 0.001), with significant declines in ghrelin, GLP-1, glucagon, leptin, PAI-1, and NEFA. These early hormonal shifts suggest that SG influences key regulators of obesity and metabolic health, though mechanisms may differ from other bariatric procedures with malabsorptive components [56].

As post-prandial hypoglycemia (PPHG) is a common adverse effect in individuals undergoing MBS [57,58] research has also been conducted to assess the hormonal changes following PPHG. In a recent case-control study, 24 individuals with pre-operation T2D who underwent RYGB were studied; half of them developed PPPHG following operation [59]. It was shown that individuals who developed PPHG had enhanced glucose clearance, due to higher insulin sensitivity and early hyperinsulinemia. Importantly, individuals with PPHG had a defective glucagon response, which prevented an increase in endogenous glucose production to prevent the hypoglycemic event [59].

The hormonal changes observed post-surgery, including alterations in glucagon, contribute to improved glycemic control and metabolic health. These findings underscore the importance of glucagon in the complex hormonal adaptations induced by MBS, supporting its role in the successful management of obesity and T2D.

#### 5.2.3. Somatostatin (SS)

SS is a peptide hormone that plays a pivotal role in regulating the endocrine system by inhibiting the release of various secondary hormones. It interacts with G protein-coupled SS receptors, influencing neurotransmission and cell proliferation [60,61]. In the pancreas, stomach, and duodenum, delta cells produce SS, which exists in two biologically active forms, 14 and 28 amino acids long, resulting from the cleavage of a pre-prohormone at distinct sites. In addition to its role in the central nervous system, SS has significant effects within the gastrointestinal tract and other endocrine organs. Specifically, SS reduces the secretion of key gastrointestinal hormones, including gastrin, secretin, CCK, GIP, and GLP-1. In the pituitary gland, it suppresses the secretion of prolactin, thyroid-stimulating hormone, and growth hormone. Additionally, in the pancreas, SS inhibits exocrine secretion and reduces the production and release of glucagon and insulin. In preclinical studies involving rats on a high-fat diet, the SS analogue octreotide, which shows a high binding affinity for SS receptor 2 and a lower affinity for receptors 3 and 5, has been observed to decrease adiposity [60,61]. However, the role of SS following MBS remains inadequately characterized in humans, with current knowledge primarily derived from preclinical data [62]. Further research is warranted to elucidate its function and therapeutic potential in the context of bariatric interventions.

#### 5.2.4. Pancreatic Polypeptide (PP)

PP is a polypeptide expressed primarily in the head of the pancreas by PP cells in the endocrine pancreas [63]. It has 36 amino acids. In addition to its effects on hepatic glycogen levels and gastrointestinal secretions, PP self-regulates the endocrine and exocrine secretion activities of the pancreas. Its secretion is lowered by SS and intravenous glucose, and it is elevated in humans following a protein meal, fasting, exercise, and acute hypoglycemia [63].

A study by Dixon et al. assessed whether the structurally related satiety hormones PYY and PP affected the overall percentage of weight loss following AGB [64]. A cross-sectional study looked at 17 postoperative individuals who had already lost an average of 28% of their body weight due to laparoscopic AGB. Prior to laparoscopic AGB, 16 people with obesity participated in a prospective study that evaluated their plasma PP and PYY meal responses. Higher weight loss was associated with lower fasting PYY levels (*p* = 0.02) and lower PP meal responses (*p* = 0.01). After an average of 53 months, the prospective study found that the subsequent mean weight loss was 20%. Significantly greater weight loss after AGB was predicted by a low preoperative PP meal response (2 h area under the curve (AUC)) (*p* = 0.024). Eight people who responded to PP meals the least lost more weight than eight who responded to PP meals the most (median 25% vs. 14%, *p* = 0.004). The mean PP meal responses were the same for all three groups. However, compared to the pre-operative group or the BMI-matched controls, the postoperative group’s fasting PYY levels were significantly lower (*p* = 0.03). After AGB, PYY seems to decrease proportionately to weight loss, which could indicate an attempt at orexigenic homeostatic compensation. Low PP meal response may indicate greater weight loss, even though PP responses seem to be unaffected by weight loss status. A person’s susceptibility to the mechanism of AGB-induced weight loss may be predicted by their PP meal response, a biological marker [64].

#### 5.2.5. Vasoactive Intestinal Polypeptide (VIP)

VIP is a 28-amino acid neuropeptide and neurotransmitter that is broadly distributed throughout the central and peripheral nervous systems [65]. Both neurons and immune cells synthesize and release VIP, which interacts with specific receptors expressed on immune and various other cell types. VIP exhibits a diverse range of biological effects, acting as a neurotransmitter, vasodilator, secretagogue, and immune regulator [65]. A study assessed the impact of RYGB on the metabolic response to orally administered glucose, specifically examining levels of glucose, insulin, VIP, neurotensin, and motilin in eight morbidly individuals with obesity, both pre- and postoperatively [66]. Prior to the surgery, an oral glucose tolerance test (OGTT) demonstrated hyperinsulinism and glucose intolerance in all individuals who remained asymptomatic throughout the test. Notably, the plasma levels of VIP, neurotensin, and motilin were undetectable during this initial assessment. However, three months post-RYGB, after an average 21% weight loss, all individuals experienced acute symptoms during repeat OGTT, including facial flushing, palpitations, nausea, abdominal fullness, pallor, diaphoresis, vomiting, and diarrhea. Importantly, three of the eight individuals exhibited an elevated release of VIP, while seven showed a significant increase in neurotensin levels. These findings suggest that the hormonal changes associated with RYGB may be linked to the pathophysiology of dumping syndrome, underscoring the role of VIP and neurotensin in postoperative metabolic responses [66]. In conclusion, this study highlights the significant hormonal shifts that occur after RYGB, contributing to the understanding of metabolic adaptations and potential complications such as dumping syndrome in individuals with obesity.

### 5.3. Small and Large Intestine Hormones

#### 5.3.1. Glucagon-like Peptide 1 (GLP-1)

GLP-1 is a peptide hormone with 30 amino acids that is produced when the proglucagon gene undergoes tissue-specific posttranslational processing [67,68]. After eating, it is created and released by intestinal enteroendocrine L-cells and specific neurons in the brain stem’s solitary tract nucleus. GLP-1 is the only incretin known to have the capacity to lower blood sugar levels in a glucose-dependent manner by increasing insulin secretion, in addition to GIP [67,68]. GLP-1 has been linked to a variety of regulatory and protective effects in addition to its insulinotropic effects [69,70,71,72]. However, a common observation following MBS in both humans and animal models is the sharp increase in a number of gut peptides, such as satiety GLP-1, after meals. Weight loss and improvements in glucose metabolism following surgery have been attributed primarily to an increase in endogenous GLP-1 signaling. But there is ongoing discussion about how much GLP-1 and other gut peptides contribute to the metabolic improvements following MBS [69,70,71,72].

Following RYGB, individuals with T2D experience improved β-cell function primarily driven by increased GLP-1 secretion. A study by Jørgensen and colleagues [73] showed that after surgery, GLP-1 levels and β-cell glucose sensitivity doubled. Using the GLP-1 receptor antagonist exendin (9–39), they found that, before surgery, blocking GLP-1R had no effect, but after RYGB, blockade reduced glucose tolerance and normalized β-cell sensitivity, emphasizing GLP-1’s critical role in metabolic improvements [73]. Another study revealed that RYGB raised post-meal GLP-1 levels over three times higher than AGB and controls, correlating with increased insulin secretion and lower glucose. This indicates that an enhanced incretin response underpins better glucose regulation and weight loss [74]. A third study examined islet responsiveness and found that RYGB increased postprandial GLP-1 and insulin secretion and improved insulin sensitivity at 1 week and 3 months [75]. Despite hormonal changes, responses to intravenous GIP and GLP-1 remained unchanged, but OGTT showed paradoxical glucagon rise and decreased 2-h glucose, linking hormonal adaptations with improved glycemic control [75]. These findings highlight that RYGB enhances endogenous GLP-1 secretion and β-cell responsiveness, which play a central role in improving glucose homeostasis and promoting remission of T2D.

#### 5.3.2. Glucagon-like Peptide 2 (GLP-2)

GLP-2 is a 33-amino acid peptide that is generated from the posttranslational proteolytic cleavage of proglucagon, a process that also produces GLP-1 [76,77]. GLP-2 is synthesized by intestinal endocrine L cells and numerous neurons in the central nervous system, and both GLP-2 and GLP-1 are co-secreted in response to nutrient ingestion [76,77]. A study aimed to investigate the relationship between satiety regulation after RYGB and the levels of GLP-1 and GLP-2 [78]. Eleven participants who had undergone RYGB were monitored over the course of one year. Satiety was assessed using a visual analogue scale (VAS) to correlate scores with the levels of GLP-1 and GLP-2 measured pre- and post-surgery. The results indicated a significant increase in the AUC for GLP-2 following the standard meal tolerance test, from 945.3 ± 449.1 to 1787.9 ± 602.7 (*p* = 0.0037). Notably, post-surgery, the AUC for GLP-1 showed a significant negative correlation with the VAS response to the question “How hungry do you feel?” (*p* = 0.008). In contrast, the AUC for GLP-2 demonstrated a significant positive correlation with the mean scores for two additional VAS questions: “How full do you feel?” and “How much do you think you can eat?” (*p* = 0.005 and *p* = 0.042, respectively). These findings suggest that both GLP-1 and GLP-2 are significantly associated with satiety assessment in this sample of individuals following RYGB, providing insight into the hormonal mechanisms underlying appetite regulation after MBS [78].

#### 5.3.3. Cholecystokinin (CCK)

Another gastrointestinal peptide hormone, CCK plays crucial roles in metabolic physiology, maintaining a healthy nutritional state, and possibly preventing and treating obesity, which is currently one of the leading causes of direct or indirect morbidity and mortality [79]. A comparative study of RYGB and SG over one year in non-diabetic individuals showed that both procedures resulted in significant weight loss and improved insulin sensitivity [80]. Post-surgery, both groups had increased postprandial GLP-1 and PYY levels, contributing to improved glucose control. RYGB ghrelin levels returned to preoperative values by 12 months, restoring normal fluctuations, while SG individuals maintained suppressed ghrelin levels. CCK responses differed: they increased less after RYGB but were significantly higher in SG, indicating different hormonal adaptations beyond the anatomical bypass effects [80]. Another study [81] investigated factors affecting weight loss responses after RYGB. Good responders displayed greater GLP-1 secretion, more ghrelin suppression, and reduced hunger during meals, while poor responders had higher CCK secretion and less favorable hormone responses. Exaggerated gut hormone release correlated with better weight loss, emphasizing the role of hormonal regulation in surgical success [81]. A third study assessed SG’s effects on hormone responses, appetite, and gastric motility. The results showed that SG individuals had lower fasting ghrelin, faster gastric emptying, and higher postprandial GLP-1 and CCK levels, leading to increased satiety and reduced hunger. These hormonal shifts, along with improved insulin sensitivity, likely drive weight loss and better glucose control post-SG [82]. These data demonstrate that hormonal changes, including decreased ghrelin, increased GLP-1 and CCK, and altered gastric emptying, are central to weight loss and metabolic improvements following MBS [82]. Understanding these hormone dynamics is key to optimizing treatment outcomes.

#### 5.3.4. Pancreatic Peptide YY

In humans, the gene encoding PYY, also known as PYY3-36, has been extensively studied for its role in appetite regulation [83,84,85]. PYY is a 36-amino acid peptide secreted by L enteroendocrine cells located in the distal small intestine and colon in response to food intake. After its release, PYY1–36 is rapidly converted to PYY3–36 in the bloodstream by the enzyme dipeptidylpeptidase-IV (DPP-IV). PYY3–36 is believed to enhance feelings of satiety by activating the Y2 receptor in the brain. The effects of PYY3–36 are multifaceted; it is known to slow gastric emptying, decrease postprandial insulin secretion, and modulate colonic motility. However, its primary role appears to be the central regulation of appetite [83,84,85]. Research conducted by Le Roux et al. demonstrated that individuals with obesity exhibit significantly lower postprandial levels of PYY3–36 compared to those of normal weight. Furthermore, studies have shown that infusion of PYY3–36 leads to reduced food intake in participants, suggesting that enhancing PYY signaling could have therapeutic implications for appetite control and weight management [86]. In summary, PYY3–36 plays an essential role in mediating satiety and appetite regulation, with potential applications in the treatment of obesity and related metabolic disorders. Understanding the mechanisms governing PYY secretion and action may offer new avenues for therapeutic interventions aimed at improving appetite control.

#### 5.3.5. Oxyntomodulin (OXM)

OXM originates from the proglucagon gene through alternative posttranslational processing pathways [87]. It is a naturally occurring 37-amino acid peptide structurally similar to glucagon, with an additional C-terminal octapeptide [88]. OXM is produced by the L cells in the colon and has been found to suppress appetite. Glucagon receptors and GLP1 receptors are activated by OXM, exhibiting a more dynamic range of effects that are more likely to correspond with changing health gain objectives in the treatment of obesity [88]. Data indicate that gut hormones significantly contribute to the metabolic improvements seen after MBS. One study [89] found that RYGB enhances the OXM response to glucose, more than doubling its levels post-meal, and this increase correlates with higher GLP-1 and PYY levels. These changes occur early and are independent of weight loss, suggesting that gut hormone reprogramming drives improved glucose homeostasis, glucose tolerance, and appetite regulation [89]. Another study observed that, within days after RYGB, there were increased postprandial levels of GLP-1, OXM, and insulin sensitivity, alongside a decrease in fasting glucose, reduced insulin resistance, and improved satiety. Over two months, the amino acid and glucose absorption reflected these hormonal shifts, with faster gastric emptying and greater insulin efficiency supporting better glucose control and weight loss [90]. A long-term study tracked RYGB and SG individuals over 10 years. The results showed sustained weight loss, increased lean muscle mass, and continued improvements in metabolic, cardiovascular, and lipoprotein profiles. Notably, SG individuals maintained lower ghrelin levels, while RYGB resulted in increased secretion of proglucagon-derived peptides like glicentin and OXM. The persistent hormonal changes, especially in gut-derived hormones, underpin the enduring health benefits seen long after surgery, including reduced cardiometabolic risk and stable weight management [91]. The evidence suggests that alterations in gut hormones, particularly increased GLP-1, OXM, and PYY, along with suppressed ghrelin, are fundamental to the long-term weight loss, improved glucose regulation, and cardiovascular health observed after MBS. These hormonal shifts are critical mechanisms driving durable metabolic benefits.

### 5.4. Duodenum Hormones

#### 5.4.1. Glucose-Dependent Insulinotropic Polypeptide (GIP)

GIP is derived from a 153-amino acid proprotein encoded by the GIP gene and circulates as a biologically active 42-amino acid peptide [92,93]. GIP, a substance with similar effects to GLP-1, is produced by K cells situated within the mucosa of the duodenum and jejunum in the digestive system. Consumption of oral glucose triggers a response, often referred to as the incretin effect, which involves an increase in insulin secretion [92,93]. A study aimed to clarify the mechanisms underlying diabetes reversibility following malabsorptive MBS [94]. Nine individuals with obesity and T2D had their peripheral insulin sensitivity and β-cell function evaluated before and one month after biliopancreatic diversion using minimal model analysis and either intravenous (IVGTT) or OGTT tests. The results were compared to those of six normal-weight control subjects. GIP, GLP-1, and the euglycemic clamp were used to measure insulin-dependent whole-body glucose disposal. Following the procedure, the IVGTT’s initial phase of insulin secretion completely returned to normal. The disposition index from IVGTT data increased roughly 3.5 times, as was the case following the euglycemic clamp, and the disposition index from OGTT data increased roughly 10 times, becoming comparable to the values observed in the control subjects. For GIP, the AUC dropped by approximately four times, *p* < 0.05. The AUC for GLP1 nearly tripled, *p* < 0.001. Changes in GIP or GLP1% and adjustments to the sensitivity indices, regardless of the glucose administration route, did not correlate significantly. In individuals with T2D, the restoration of first-phase insulin secretion and normalization of insulin sensitivity following malabsorptive MBS appears to be associated with a decrease in the impact of some intestinal factors or factors brought on by intestinal bypass [94].

Researchers assessed early alterations in insulin physiology, gut hormone responses, and glycemia after AGB or RYGB surgery using an easily tolerated, slowly ingested solid, low-carbohydrate MMT in a prospective non-randomized study [95]. In individuals who were metabolically healthy and with obesity (gastric banding = 8 or RYGB = 10), the MMT responses of plasma glucose, insulin, and c-peptide (to estimate hepatic insulin extraction; %HIE), incretins (GIP, aGLP-1), and PP were assessed 4–8 weeks prior to and following surgery. There was no change in %HIE (*p* = 0.98) after gastric banding surgery, but the C-peptide and insulin MMT profiles (*p* = 0.004 and *p* = 0.0005, respectively) were lower. The prandial aGLP-1 responses’ trajectory was changed by RYGB (treatment × trajectory *p* = 0.02), and PP was decreased (*p* < 0.0001). The response patterns of plasma glucose, insulin, and gut hormones to a solid, slowly ingested low-carbohydrate MMT are significantly different after RYGB than after AGB. Glycemia improved more quickly following RYGB than following AGB, which is consistent with altered nutrient delivery and indirect evidence of changes in hepatic and peripheral insulin physiology [95].

#### 5.4.2. Secretin

A low intraluminal pH causes the duodenal mucosa’s S cells to produce the 27-amino acid peptide hormone secretin, which suppresses the stomach’s parietal cells’ ability to secrete gastric acid and promotes the pancreatic centroacinar cells’ and intercalated ducts’ production of bicarbonate [96,97]. In the duodenum, it emulsifies dietary fats for the action of pancreatic lipase. It also encourages the liver to produce bile. In humans, the secretin gene encodes the secretin peptide [96,97].

A study by Modvig et al. showed that secretin levels were increased following RYGB, indicating that the surgery may enhance secretin secretion through nutrient diversion to the distal small intestine, which could contribute to improved glycemic regulation and metabolic outcomes [98]. One study examined how RYGB affects hormone gene expression and small-intestinal enteroendocrine cell density in individuals with obesity and T2D [99]. Twelve individuals underwent RYGB and were followed by enteroscopy after 10 months. The results showed increased density of cells immunoreactive for GLP-1, CCK, and GIP in post-surgery individuals, while controls showed increases in GLP-1, PYY, CCK, and PC2. Secretin, along with ghrelin and GIP mRNA levels, decreased in both groups post-surgery, while PYY, CCK, NTS, and NR1H4 gene expressions remained unchanged. GCG mRNA increased in both groups. These changes suggest that RYGB induces significant cellular and molecular alterations in enteroendocrine hormones, particularly increasing GLP-1, PYY, CCK, and GIP-producing cell populations, and reducing ghrelin, secretin, and GIP gene expression [99]. Another study assessed the effects of RYGB on enteroendocrine cells in 18 non-diabetic individuals, comparing different biliopancreatic limb lengths [100]. After 12 months, GLP-1 cell density increased nearly fivefold in the jejunum, especially in the BP200 group, with doubled PYY and GIP cell densities. While GLP-1 and PYY mRNA levels decreased, GIP mRNA remained stable. RYGB did not significantly affect the villus length or cell densities of ghrelin, CCK, neurotensin, secretin, or serotonin. The increase in incretin-producing cells likely explains elevated plasma incretin levels post-surgery [100]. Some studies demonstrate that meal-induced secretin activates brown fat thermogenesis, contributing to satiation and increased energy expenditure, highlighting brown adipose tissue’s (BAT) potential as a target for obesity treatment [101,102,103]. More research is needed. Interestingly, a recent unknown effect of secretin on activating BAT and on regulating appetite has been reported in humans.

**Figure 2:**
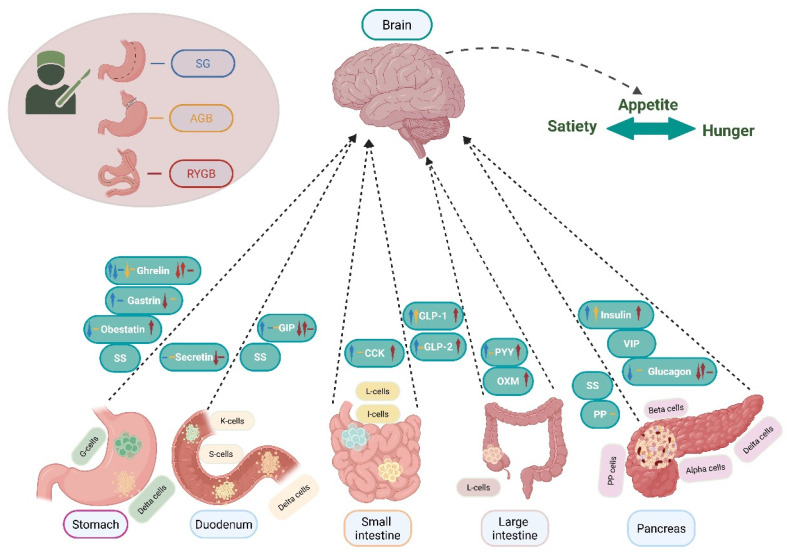
Neuroendocrine interactions between the brain and gastrointestinal organs post-MBS. Created in BioRender. Anastasiou, I.A. (2025) https://BioRender.com/a10utz8, (accessed on 13 August 2025). AGB: adjustable gastric banding; CCK: cholecystokinin; GIP: glucose-dependent insulinotropic peptide; GLP-1: glucagon-like peptide-1; GLP-2: glucagon-like peptide 2; OXM: oxyntomodulin; PP: pancreatic polypeptide; PYY: peptide YY; RYGB: Roux-en-Y gastric bypass; SG: sleeve gastrectomy; SS: somatostatin; VIP: vasoactive intestinal polypeptide.

Hormone interactions between the brain and organs of the gastrointestinal tract after MBS are depicted in Figure 2.

## 6. Fibroblast Growth Factors (FGF-19, FGF-21)

FGF-19 and FGF-21 are significant hormones that regulate metabolic processes during both feeding and fasting, subsequently influencing energy production [104,105]. While FGF-21 is produced in the liver during fasting and is necessary to regulate glucose and lipid metabolism and to maintain homeostasis, FGF-19 is excreted in the intestine during feeding and is negatively related to the synthesis and secretion of bile acids. FGF-19 and FGF-21 are considered hormone-delayed factors because they are secreted only after the actions of glucagon and insulin have been completed. Both FGF-19 and FGF-21 share overlapping functions, including enhancing glucose tolerance, improving insulin sensitivity, promoting weight loss, reducing lipids, and increasing metabolic activity. Although they are activated under different physiological conditions, their roles converge to effectively regulate metabolic processes. This is particularly true for pathologies such as T2D, obesity, metabolic syndrome, cardiovascular disease, and renal disease [104,105].

A study by Gómez-Ambrosi et al. analyzed the impact of weight loss induced either by conventional dietary treatment or MBS on FGF-19 and FGF-21 concentrations [106]. Serum concentrations of FGF-19 and FGF-21 were measured in 137 individuals with obesity and different degrees of insulin resistance matched by sex, age, and body adiposity and compared to 33 lean individuals. Furthermore, FGF-19 and FGF-21 were measured in 114 subjects before and one year after weight loss induced either by conventional dietary treatment (N = 28), SG (N = 20), or RYGB (N = 66). Circulating serum FGF-19 concentrations were similarly lower (*p* < 0.01) in individuals with obesity, regardless of their degree of insulin resistance, while FGF-21 levels were higher in individuals with obesity (*p* < 0.01), and further increased in obesity-associated T2D (*p* < 0.01) compared to healthy controls. FGF-19 concentrations were increased in subjects with obesity after surgically induced weight loss (*p* < 0.01), but not after weight loss achieved by conventional dietary treatment, while FGF-21 levels were reduced after conventional dietary treatment (*p* < 0.05) or SG-induced weight loss (*p* < 0.05), but not after RYGB. The change in FGF-21 concentrations emerged as a significant predictor of the change in HOMA after weight loss. It was concluded that FGF-21 is primarily related to glucose homeostasis, while FGF-19 is primarily related to body weight, specifically visceral adipose tissue, based on circulating concentrations and the subsequent pattern of response after weight loss [106].

In another study, researchers investigated the effect of different bariatric procedures on circulating FGF-19 levels and the resulting impact on mitochondrial health in white adipose tissue [107]. T2D women with obesity (n = 39, BMI > 35 kg/m^2^) undergoing either BPD, laparoscopic greater curvature plication (GCP), or AGB participated in the study. Anthropometry, biochemical, clinical data, serum, and adipose tissue biopsies were collected before, and 6 months after surgery. Mitochondrial gene expression in adipose biopsies and serum FGF-19 levels were then assessed. All types of MBS led to metabolic improvements, with BPD producing the greatest benefits on weight loss, hemoglobin A1C (HbA1c), and cholesterol reduction, while GCP resulted in similar HbA1c improvements (adjusted for BMI). Circulating FGF-19 increased in both BPD and GCP (*p* = 0.018), while, in AGB, FGF-19 serum levels decreased (*p* = 0.028). Interestingly, circulating FGF-19 levels were inversely correlated with the mitochondrial number in adipose tissue across all surgeries (n = 39). In contrast to GCP and AGB, the mitochondrial number in BPD individuals corresponded directly with changes in 12 of 14 mitochondrial genes assayed (*p* < 0.01). Following surgery, elevated serum FGF-19 levels were linked to both T2D remission and better mitochondrial health in adipose tissue. Among the study procedures, BPD produced the best metabolic outcomes (BPD > GCP > AGB), indicating that FGF-19 may target mitochondria in adipose tissue during T2D remission [107].

In another study, researchers investigated the roles of FGF-19, FGF-21, and total bile acid among those with morbid obesity and T2D undergoing RYGB [108]. A total of 35 individuals were enrolled. Plasma FGF-19, FGF-21, and total bile acid levels were measured before surgery, 3 months, and 12 months after surgery, while the hepatic steatosis index was calculated before and after surgery. Individuals with T2D and obesity after RYGB presented with increased serum FGF-19 levels (*p* = 0.024) and decreased total bile acid (*p* = 0.01) and FGF-21 levels (*p* = 0.005). Individuals with T2D who achieved complete remission had a higher FGF-19 level at 3 months (*p* = 0.004) compared with individuals with T2D who did not achieve complete remission. Fatty liver improvers tended to have lower FGF-21 levels (*p* = 0.05) compared with non-improvers at 12 months. Following RYGB, the remission of T2D and improvement in metabolic dysfunction-associated steatotic liver disease (MASLD) in individuals are affected differently by changes in FGF-19 and FGF-21. A decrease in serum levels of FGF-21 may indicate improvement in MASLD following RYGB, while an early increase in serum levels of FGF-19 may indicate complete remission of T2D [108]. Table 1 summarizes studies examining gastrointestinal hormonal changes following metabolic bariatric surgery, categorized by the type of surgical procedure.

## 7. Endocrine and Reproductive Outcomes of Metabolic Bariatric Surgery

A comprehensive review adhering to PRISMA guidelines was undertaken to analyze peer-reviewed studies examining endocrine parameters before and after MBS in humans [109]. Selected for their relevance and methodological quality, these studies highlight the complex endocrine effects of MBS. While MBS can restore growth hormone secretion and enhance fertility, it may also disrupt insulin-like growth factor-1 recovery and disturb sex hormone homeostasis, potentially resulting in bone loss and a catabolic state. Postprandial hyperinsulinemia can cause PPHG, often accompanied by impaired counter-regulatory hormone responses. Additional risks include secondary hyperparathyroidism and decreased bone mineral density. Changes in thyroid hormone levels further complicate the endocrine landscape, affecting both hypothyroid and euthyroid individuals. These findings reflect the delicate balance between metabolic improvements and the emergence of endocrine dysfunction. Most existing evidence consists of associative studies that lack the robustness needed for definitive clinical guidance, underscoring the urgent need for high-quality research to establish causal relationships and inform best practices. Addressing the underlying mechanisms driving these endocrine alterations is essential for optimizing MBS outcomes [109]. A comprehensive approach encompassing thorough preoperative assessment, personalized postoperative management, and targeted therapeutic interventions is critical to minimizing complications while maximizing the benefits of MBS.

MBS has shown significant potential in improving sexual function and fertility, though these effects often vary between genders [110]. In men, enhancements in sexual performance are typically linked to weight reduction and normalization of sex hormone levels. Conversely, improvements in female sexual function are influenced by a broader spectrum of factors beyond mere weight loss. A prospective study examined the long-term effects of SG on hormonal profiles, sperm parameters, and sexual function in 54 men with obesity and infertility [111]. The results showed that weight loss was associated with improvements in lipid profiles and significant increases in sex hormone-binding globulin, total, and free testosterone levels at 12 and 18 months post-surgery. Sperm count and total sperm number also improved significantly, though other semen parameters remained unchanged. Additionally, sexual desire, erectile function, and satisfaction were markedly enhanced after surgery. Overall, SG effectively improves hormonal balance, sperm quality, and sexual function in infertile men with obesity [111]. While MBS has shown limited or even negative effects on male fertility, sometimes reducing sperm count and quality, it appears to have more favorable outcomes for female reproductive health, including increased pregnancy rates and better neonatal results. A prospective study evaluated 67 women undergoing MBS to assess its effects on hormonal and clinical reproductive parameters [112]. The results showed significant weight and BMI reduction, along with decreases in androstenedione and testosterone levels. Conversely, levels of sex hormone-binding globulin, dehydroepiandrosterone sulfate, and anti-müllerian hormone increased. Clinical improvements included reduced severity of dysmenorrhea and a decrease in hirsutism and polycystic ovary morphology at 6 months postoperatively. The findings indicate that MBS has a beneficial effect on hormonal regulation and reproductive health in women of reproductive age with obesity [112]. Nonetheless, due to risks associated with malnutrition and rapid weight loss, it is generally recommended to delay conception for 12 to 18 months post-surgery to optimize maternal and fetal safety [110].

A comprehensive systematic review evaluated 34 studies on the impact of MBS on female reproductive health [113]. The findings indicate that over 75% of studies reported improved ovulatory and menstrual function, increased pregnancy rates, and better neonatal outcomes following surgery. Notably, post-surgical pregnancies were linked to lower incidences of gestational diabetes, hypertensive disorders, and macrosomia. Nonetheless, some studies have highlighted potential nutritional deficiencies and risks of low birth weight, especially after RYGB. It is advisable to postpone conception for 12 to 18 months post-surgery to enhance safety and outcome quality [113]. The variability across studies underscores the need for standardized research methodologies. Overall, MBS offers significant benefits for female fertility, particularly in women with polycystic ovary syndrome, but demands careful preconception counseling, nutritional monitoring, and multidisciplinary care. Future research should focus on long-term reproductive health, establishing uniform assessment protocols and developing comprehensive guidelines for pregnancies after bariatric procedures.

## 8. Current Evidence and Challenges in Comparing Surgical Techniques for Obesity and Metabolic Outcomes

Among the numerous MBS techniques available, the two most commonly utilized due to their superior efficacy and safety profiles are RYGB and SG. Despite extensive research in this field, there are relatively few randomized controlled trials (RCTs) addressing these procedures. Furthermore, the inherent difficulties in conducting large multicenter RCTs in this domain imply that acquiring robust, definitive evidence will likely remain elusive in the foreseeable future. Although several prior RCTs have evaluated metabolic outcomes such as weight reduction and the rate of T2D remission, these studies have generally focused on comparative efficacy rather than the underlying physiological mechanisms. Since the main goal of MBS is to treat T2D, it is clinically relevant whether one operation outperforms the other. SLEEVEPASS was a multicenter, open-label, randomized clinical equivalence trial, where 240 individuals with severe obesity were randomly assigned to either SG or RYGB [114]. Both operations induced complete or partial remission of T2D to a similar extent [114]. The same conclusion was reached when the SLEEVEPASS and SM-BOSS data were combined [115].

Although several previous RCTs have compared metabolic outcomes in terms of weight loss and T2D remission across the various bariatric procedures, to the best of our knowledge, there are no RCTs that have assessed as primary outcomes differences in surgeries in the hormonal responses [116].

A systematic review evaluated RCTs comparing at least two surgical options such as RYGB, AGB, and SG, for severe and complex obesity [116]. The assessment employed the PRagmatic Explanatory Continuum Indicator Summary-2 (PRECIS-2) to gauge how well the study designs reflect routine clinical practice, and the Risk of Bias 2 (RoB 2) tool to determine methodological validity. Searches in MEDLINE, Embase, and the Cochrane Central Register of Controlled Trials (CENTRAL) identified 15 relevant studies published between November 2013 and June 2021.

According to the PRECIS-2 evaluation, three studies were classified as pragmatic, offering findings that are highly applicable to everyday clinical settings. Ten studies demonstrated a combination of pragmatic and explanatory features, often due to design elements such as being single-center, having fixed intervention methods, or involving intensive follow-up regimens. Only two trials achieved a low risk of bias, with one of these also being pragmatic. Conversely, three studies carried a high risk of bias, limiting confidence in their results.

Overall, few MBS trials combine strong methodological quality with a pragmatic approach. There is an urgent need for high-quality, pragmatic research to provide clear, applicable evidence that can effectively guide clinical practice and optimize patient outcomes [116].

## 9. Limitations, Challenges, and Future Perspectives

Limitations in the current research on MBS’s metabolic and endocrine outcomes are significant. Most available studies are observational, rendering them susceptible to biases and confounding factors, with only a limited number of RCTs providing robust, high-quality data, particularly those focused on hormonal responses as primary outcomes. The variability in surgical techniques, patient selection criteria, and follow-up durations further complicates the ability to make direct comparisons across studies, hampering efforts to establish standardized protocols and evidence-based guidelines. Additionally, many studies emphasize only immediate or short-term results, leaving a gap in knowledge regarding long-term metabolic, hormonal, and safety profile factors that are crucial for comprehensive patient management. Methodological challenges, including ethical issues associated with large multicenter RCTs, hinder the generation of definitive comparative data, especially concerning physiological and hormonal mechanisms.

Ensuring consistent methodologies, patient adherence, and long-term follow-up remains a significant hurdle. Moreover, understanding the underlying hormonal and physiological mechanisms responsible for the metabolic benefits observed after surgery is essential for optimizing surgical techniques, but current studies frequently lack detailed mechanistic insights. Personalizing surgical interventions based on individual hormonal, metabolic, and genetic profiles continues to be a challenge due to limited comprehensive data. Additionally, monitoring long-term safety poses difficulties, as adverse effects such as nutritional deficiencies, bone loss, and metabolic disturbances require extended observation periods, which many studies fail to provide.

Looking at the future, research should focus on developing standardized, pragmatic, multicenter trials that closely resemble routine clinical practice, making the findings directly applicable to patient care. A greater emphasis on mechanistic studies exploring hormonal, microbiome, and metabolic pathways will deepen the understanding of how different procedures influence health outcomes. Furthermore, integrating genetic, hormonal, and metabolic profiling into preoperative assessment could pave the way for personalized surgical strategies, thereby improving efficacy and reducing complications. Extended long-term follow-up studies are essential to assess the durability of benefits and identify potential adverse effects, enabling the development of safer and more effective treatment algorithms. The identification of non-invasive biomarkers capable of predicting individual responses to surgery will help optimize patient selection and postoperative management.

In recent years, due to the use of GLP-1 receptor agonists and of tirzepatide, referral of individuals with severe obesity to MBS is expected to be reduced or delayed. This clinical decision may affect the progress of our better understanding of hormonal modification following MBS. The challenge of MBS researchers will be to conduct large, well-designed prospective studies, with contemporary evaluation not only of the metabolic outcomes, but also of the pathophysiological players contributing to them. Co-financing for MBS studies is essential to this end. Although the IFSO has recently created a global registry, this includes information on demographics, information regarding diseases related to obesity, and perioperative outcomes [117].

To optimize the evolving landscape of MBS, future efforts should prioritize standardized, pragmatic, multicenter trials that enhance the direct applicability of findings to patient care; a greater emphasis on mechanistic studies exploring hormonal, microbiome, and metabolic pathways; future investigations integrating genetic, hormonal, and metabolic profiling into preoperative assessments to enable personalized surgical strategies; extended long-term follow-up studies to assess the durability of benefits and identify potential adverse effects; identification of non-invasive biomarkers to optimize patient selection and postoperative management, proactively addressing the expected reduction or delay in referrals for MBS and conducting studies, fully utilizing global registries, and finally, adopting a multidisciplinary, patient-centered approach combining surgical, nutritional, hormonal, and psychological care to be vital in achieving better outcomes and higher patient satisfaction.

## 10. Clinical and Translational Relevance

MBS offers significant clinical benefits, particularly in the management of diabetes, as it improves insulin sensitivity and beta-cell function, often leading to diabetes resolution. However, meticulous patient selection and careful management are essential to achieve optimal outcomes. MBS also influences gut hormones, such as ghrelin, GLP-1, and PYY, resulting in decreased appetite and weight loss. Understanding these hormonal mechanisms is crucial for effectively managing patient expectations and providing appropriate postoperative care. Different surgical procedures, like RYGB and SG, evoke distinct hormonal responses, which should be considered when planning personalized surgical strategies. Vigilance for endocrine complications such as cachexia is necessary, emphasizing the importance of thorough preoperative evaluation and ongoing postoperative monitoring. Additionally, MBS positively affects hormonal balance and fertility in both men and women, warranting counseling regarding potential reproductive benefits.

From a translational perspective, insights gained from hormonal changes post-MBS are guiding the development of less invasive obesity treatments and enabling targeted interventions to improve weight loss and glycemic control. Personalizing surgical approaches based on individual hormonal profiles and identifying predictive biomarkers may optimize patient outcomes. Further research into the complex interactions among hormones, the microbiome, and genetics is vital for enhancing long-term success and advancing future therapeutic strategies.

## 11. Conclusions

While our understanding of hormonal changes after MBS continues to develop, several key clinical applications are emerging. Hormonal markers such as sustained postprandial GLP-1 and suppressed ghrelin levels may serve as indicators of surgical success and metabolic improvement. Monitoring these hormones postoperatively may guide personalized dietary recommendations and supplementation, and future insights could inform surgical decisions and the development of drugs that mimic beneficial hormonal effects. However, responses like gastrin secretion vary depending on the procedure, and the long-term impact of hormonal changes remains unclear.

Future research should prioritize mechanistic, individual-level studies to distinguish hormonal effects from weight loss and assess long-term outcomes. Although collecting detailed hormonal data is challenging due to privacy regulations, it is essential for understanding how these responses influence overall health and the success of MBS. This knowledge could enable the development of more targeted, less invasive treatments and improve personalized care for obesity and related metabolic disorders.

## Figures and Tables

**Table 1:** Studies on gastrointestinal hormonal changes following metabolic bariatric surgery, according to the surgical procedure.

Author, Year	Surgery Procedure	Participants	Methodology	Follow-up Duration	Hormones Assessed	Key Outcomes
Roux-en-Y gastric bypass						
Cummings et al., 2002 [36]	RYGB	13 pre-/post-diet, 5 post-RYGB, 10 controls	Plasma hormones	Not specified	Ghrelin	Ghrelin increases with diet-induced weight loss
Hedberg et al., 2005 [28]	RYGB	6 individuals with endoscopically confirmed stomal ulcer (2–6 yrs post), 6 controls	Endoscopy and clinical	2–6 years	Gastric acid markers	Gastric acid involved in stomal ulcers
Laferrère et al., 2010 [89]	RYGB	20 women with T2D	Glucose and hormone profiling	Not specified	GLP-1, PYY, OXM	Hormone peaks correlated with diabetes remission
Falkén et al., 2011 [90]	RYGB	12 individuals with obesity	Postprandial hormones	3 days, 2 months, and 1 year	GLP-1, OXM	Postprandial increases promote weight loss and insulin sensitivity
Umeda et al., 2011 [55]	RYGB	10 individuals with T2D	Hormonal profiling	7, 30, and 90 days	Ghrelin, GLP-1, PYY	Early hormonal changes support glycemic control
Dirksen et al., 2013 [81]	RYGB	16 good responders, 17 poor responders, 8 controls	Meal-induced hormones	1 week and 3 months	Various gut hormones	Favorable hormonal responses support weight loss in good responders
Safatle-Ribeiro et al., 2013 [31]	RYGB	35 long-term (>36 months), 32 controls	Histological analysis	>36 months	Gastric mucosal markers	Down-regulated apoptosis and increased proliferation post-RYGB
Jørgensen et al., 2013 [73]	RYGB	9 individuals with T2D	β-cell and glucose response tests	1 week and 3 months	GLP-1, Glucagon	GLP-1 enhances β-cell function and gluc
Dirksen et al., 2013 [75]	RYGB	11 severely glucose-tolerant obese	OGTT with hormonal analysis	1 week and 3 months	Glucagon, Plasma glucose	Hypersecretion of glucagon; reduction in 2-h plasma glucose during OGTT
Camastra et al., 2013 [49]	RYGB	12 individuals with T2D, 15 non-diabetic	Postprandial tests, insulin sensitivity	1 year	Insulin, glucose, gut hormones	Improved insulin sensitivity; altered endogenous glucose output
Bojsen-Møller et al., 2014 [50]	RYGB	10 individuals with T2D, 10 non-T2D	Oral glucose tests	1 week, 3 months, and 1 year	Insulin, gut hormones	Increased insulin secretion in T2D after RYGB emphasizing gut’s role
Rhee et al., 2015 [99]	RYGB	12 individuals with T2D, 11 controls	Enteroendocrine cell profiling	~10 months	Various gut hormones	Altered enteroendocrine cell distribution and hormone gene expression
Nergård et al., 2015 [100]	RYGB	18 individuals with obesity without DM	Jejunum cell density analysis	12 months	Incretin hormones	Increased density of incretin-producing cells in jejunum
Guo et al., 2022 [108]	RYGB	35 individuals with T2D/obesity	Post-surgery FGF analysis	3 and 12 months	FGF-19, FGF-21	Changes in FGF-19/FGF-21 explain remission of DM and MASLD; shift in serum levels indicates metabolic improvements
Sleeve gastrectomy						
Cătoi et al., 2016 [51]	SG	20 individuals with obesity	Blood tests	7, 30 and 90 days	Insulin, HOMA-IR	Decreased insulin and HOMA-IR levels
Farey et al., 2017 [56]	SG	11 individuals with obesity, SG, 22 controls	Hormonal and metabolic analyses	3 months	Various gut hormones	Differences in weight loss mechanisms compared to other surgeries
Adjustable gastric banding						
Shak et al., 2008 [29]	AGB	24	Hormonal response	12 months	Ghrelin	AGB partially suppresses ghrelin spike during weight loss
Several techniques						
Frühbeck et al., 2004 [37]	AGB, RYGB	8 AGB, 8 RYGB, 8 controls	Hormonal profiling	6 months	Ghrelin	RYGB reduces ghrelin via fundus bypass; not due to weight loss or insulin sensitivity
Frühbeck et al., 2004 [38]	AGB, RYGB, BPD	7 AGB, 6 RYGB, 3 BPD	Cross-sectional analysis	Not specified	Ghrelin	Fundus dysfunction influences fasting ghrelin levels
Korner et al., 2007 [74]	RYGB, AGB	13 RYGB, 10 AGB	Meal tests	Not specified	GLP-1, GIP	RYGB enhances postprandial GLP-1 and GIP, improving glucose regulation
Huda et al., 2008 [45]	Post-gastrectomy	8 post-operation, 9 individuals with obesity, 9 controls	Gene and hormone expression	Not specified	Obestatin	Obestatin expression altered after gastrectomy
Salinari et al., 2009 [94]	Malabsorptive surgery	9 individuals with T2D, 6 controls	Hormonal and metabolic assessments	Not specified	Insulin, gut hormones	Better insulin sensitivity associated with gut hormonal changes
Martins et al., 2011 [46]	RYGB, Lifestyle	9 RYGB, 8 lifestyle, 9 controls	Hormonal measurements	3 years	Ghrelin, Obestatin	Elevated fasting ghrelin/obestatin post-RYGB linked to weight maintenance
Peterli et al., 2012 [80]	RYGB, SG	12 RYGB, 11 SG	Hormonal and clinical follow-up	1 week, 3 and 12 months	Ghrelin, CCK, GLP-1, PYY	Hormones influence weight loss mechanisms
Sillakivi et al., 2013 [32]	RYGB, SG	20 RYGB, 20 SG	Gastric function tests	22 months	Gastric hormones	Sleeve reduces corpus secretion, antral functions preserved
Grong et al., 2016 [30]	RYGB, SG	20 RYGB/SG, 13 controls	Gastrin secretion assessment	Not specified	Gastrin	Gastrin secretion decreased after RYGB
Bunt et al., 2017 [95]	RYGB, AGB	10 RYGB, 8 AGB	Glycemic response tests	4–8 weeks	Glucose, insulin	Faster glycemic improvement after RYGB than AGB
Gómez-Ambrosi et al., 2017 [106]	SG, RYGB	20 SG, 66 RYGB, 28 control	FGF-19 and FGF-21 levels	1 year	FGF-19, FGF-21	FGF-21 relates to glucose; FGF-19 associates with visceral fat reduction
Martinez de la Escalera et al., 2017 [107]	BPD, GCP, AGB	39 women with T2D and obesity	Metabolic and FGF-19 analysis	6 months	FGF-19, FGF-21	BPD yields the best metabolic outcomes; FGF-19 may target mitochondria in adipose tissue during remission
Lampropoulos et al., 2022 [40]	SG and RYGB/BPD	12 SG, 20 RYGB/BPD	Fasting/postprandial hormones	≥7 years	Ghrelin, GLP-1, PYY	No significant differences between groups; hormonal responses reflect ongoing weight changes
Brzozowska et al., 2023 [52]	RYGB, SG, AGB	7 RYGB, 21 SG, 11 AGB, 16 diet-controlled	Insulin resistance and HOMA-IR	12–36 months	PYY, Adiponectin	Reduced insulin resistance, increased PYY and adiponectin during weight stability
Kokkinos et al., 2024 [91]	RYGB, SG	11 RYGB, 17 SG	Hormonal secretion analysis	3, 6 and 12 months and 10 years	Glicentin, OXM, TMAO	RYGB enhances proglucagon products and raises cardiovascular risk marker TMAO

**Abbreviations**: AGB: adjustable gastric banding; BPD: biliopancreatic diversion; CCK: cholecystokinin; FGF: fibroblast growth factor; GCP: greater curvature plication; GIP: glucose-dependent insulinotropic peptide; GLP-1: glucagon-like peptide-1; GLP-2: glucagon-like peptide 2; HOMA-IR: homeostasis model assessment—insulin resistance; MASLD: metabolic dysfunction-associated steatotic liver disease; OGTT: glucose tolerance test; OXM: oxyntomodulin; PYY: peptide YY; RYGB: Roux-en-Y gastric bypass; SG: sleeve gastrectomy.

## Data Availability

No new data were created by this study.

## Abbreviations

The following abbreviations are used in this manuscript:

AGB: adjustable gastric banding; AUC: area under the curve; BAT: brown adipose tissue; BMI: body mass index; BPD-DS: biliopancreatic diversion with duodenal switch; CCK: cholecystokinin; CENTRAL: Cochrane Central Register of Controlled Trials; CKD: chronic kidney disease; CVD: cardiovascular disease; DPP-IV: dipeptidylpeptidase-IV; FGF: fibroblast growth factor; GCP: greater curvature plication; GI: gastrointestinal; GIP: glucose-dependent insulinotropic peptide; GLP-1: glucagon-like peptide-1; GLP-2: glucagon-like peptide 2; HbA1c: hemoglobin A1C; HOMA-IR: homeostasis model assessment—insulin resistance; IFSO: International Federation for the Surgery of Obesity and Metabolic Disorders; MASLD: metabolic dysfunction-associated steatotic liver disease; MBS: metabolic bariatric surgery; NEFA: non-esterified fatty acids; NIH: National Institutes of Health; OAGB: one-anastomosis gastric bypass; OGTT: glucose tolerance test; OSA: obstructive sleep apnea; OXM: oxyntomodulin; PGI: protein glycation index; PP: pancreatic polypeptide; PPHG: post-prandial hypoglycemia; PRECIS-2: PRagmatic Explanatory Continuum Indicator Summary-2; PYY: peptide YY; RCTs: randomized controlled trials; RoB 2: Risk of Bias 2; RYGB: Roux-en-Y gastric bypass; SG: sleeve gastrectomy; SS: somatostatin; T2D: type 2 diabetes; VAS: visual analogue scale; VIP: vasoactive intestinal polypeptide; WHO: World Health Organization.
